# Metabolomics Mechanism and Lignin Response to Laxogenin C, a Natural Regulator of Plants Growth

**DOI:** 10.3390/ijms23062990

**Published:** 2022-03-10

**Authors:** Yuchan Deng, Jiaqi Wang, Annan Zhang, Zhaoju Zhu, Sipei Ren, Cunli Zhang, Qiang Zhang

**Affiliations:** Shaanxi Key Laboratory of Natural Products & Chemical Biology, College of Chemistry & Pharmacy, Northwest A&F University, Yangling, Xianyang 712100, China; dengyuchan@sina.com (Y.D.); jiaqiwang719@163.com (J.W.); zhangann@nwafu.edu.cn (A.Z.); zhuzhaoju97@nwafu.edu.cn (Z.Z.); rensipei@nwafu.edu.cn (S.R.)

**Keywords:** plant growth regulator, LC-MS/MS, metabolomics, tomato, lignification, Laxogenin C

## Abstract

Laxogenin C (LGC) is a natural spirostanol deriving from plant hormone which has shown growing regulation similar to those of brassinosteroids. In the present study, LGC showed a promoting effect on tomato seed germination and seedling growth in a dose-dependent manner. We applied LC-MS/MS to investigate metabolome variations in the tomato treated with LGC, which revealed 10 differential metabolites (DMs) related to KEGG metabolites, associated with low and high doses of LGC. Enrichment and pathway mapping based on the KEGG database indicated that LGC regulated expressions of 2-hydroxycinnamic acid and l-phenylalanine to interfere with phenylalanine metabolism and phenylpropanoids biosynthesis. The two pathways are closely related to plant growth and lignin formation. In our further phenotypic verification, LGC was confirmed to affect seedling lignification and related phenylpropanoids, trans-ferulic acid and l-phenylalanine levels. These findings provided a metabolomic aspect on the plant hormone derivates and revealed the affected metabolites. Elucidating their regulation mechanisms can contribute to the development of sustainable agriculture. Further studies on agrichemical development would provide eco-friendly and efficient regulators for plant growth control and quality improvement.

## 1. Introduction

Plant growth regulators (PGRs) are special chemicals that regulate (promote or inhibit) the growth and development of plants at very low dosages [1], which play no phytotoxic roles. There have been a variety of PGRs successfully used in agriculture, and new types of plant hormones, including brassinosteroids (BRs). They are attractive due to their involvement in many physiological procedures and abilities to promote seed germination [2] and plant growth [3,4], enable plants to resist the harsh living environment [5], and facilitate pesticide metabolism [6]. Thus, PGRs, especially those with new scaffolds, need deep insights into their functions and mechanisms where the yield and quality of vegetable products are increased and higher planting efficiency is achieved.

Saponins are secondary metabolites commonly found in plants and can also be used as nutritional characterization in crops [7], generally having anti-inflammatory, antibacterial and antioxidant effects [8,9]. Among them, spirostanes share similarities in their physiological activity with BRs and have the potential to act as natural plant growth promoters. Steroidal glycosides of the spirostane series extracted from *Nicotiana tabacum L.* seeds presented growth-stimulating activities [10]. In general, low doses of exogenous auxin can promote plant growth. Meanwhile, high doses of exogenous auxins disturbed the homeostasis of plant hormones and caused plant death, for example, by altering auxin homeostasis, transport, and distribution to modulate root morphology and inhibit root growth [11]. However, most studies of spirostanes have focused on the relationship between regulatory effects and antioxidant enzymes instead of providing detailed insight into metabolic mechanisms.

Laxogenins C (LGC) and Smilaxin A (SA) are spirostanol saponins isolated from the traditional medicine plant *Smilax scobinicaulis* and have plant hormone activities similar to brassinosteroids [12]. Studies showed that laxogenin, a naturally spirostanic analogue, has a similar structure to BR skeletons and its derivatives show evident plant growth-promoting activity in radish [13]. Laxogenin glycosides also showed a high level of physiological activity in plant growth [14]. In this study, we explored the plant growth regulation of LGC and SA. On this basis, we elucidated the mechanism of LGC regulation of tomato seedling growth through LC-MS/MS metabolome investigation and clarified the target pathway affected by LGC.

## 2. Results

### 2.1. Tomato Growth Responding to LGC and SA Regulation

**Figure 1:**
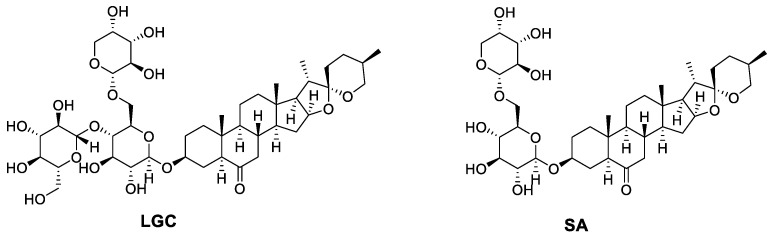
Chemical structures of LGC and SA.

The molecular formulae of SA and LGC were determined based on high-resolution mass spectrometry, and their chemical structures were determined by NMR, which were compared with those reported data [15]. Their chemical structures are shown in Figure 1.

**Figure 2:**
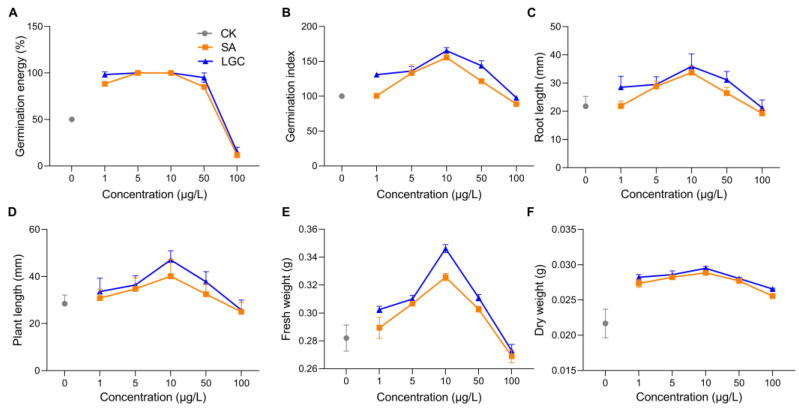
LGC and SA on germination energy (**A**), germination index (**B**) as well as seedling root length (**C**), plant length (**D**), fresh weight (**E**), and dry weight (**F**). Data are mean ± SD. (**A**,**B**,**E**,**F**), *n* = 3; (**C**,**D**), *n* = 60.

The regulation of tomato seed germination and seedling growth by different concentrations of SA and LGC are shown in Figure 2. The GE (Figure 2A) peaked at 5 μg/L and 10 μg/L when the seeds were treated with LGC and SA, respectively. At the same time, the GI (Figure 2B) peaked at 10 μg/L. Low concentrations (5 μg/L and 10 μg/L) of SA and LGC promoted seed germination. However, LGC was more effective than SA in promoting germination. Furthermore, the daily germination percentage of tomato seeds (as shown in Supplementary Figure S1) demonstrated that SA and LGC at low concentrations (1~10 μg/L) caused early seed germination and delayed seed germination at increasing concentrations.

The growth of plants can be evaluated by root length (Figure 2C), plant length (Figure 2D), fresh weight (Figure 2E), and dry weight (Figure 2F) of seedlings. These data showed the same trend peaking at a concentration of 10 μg/L and then decreasing. These effects were also evident in the morphology observation, as shown in Supplementary Figure S2.

### 2.2. LC-MS/MS Metabolome Analysis

**Figure 3:**
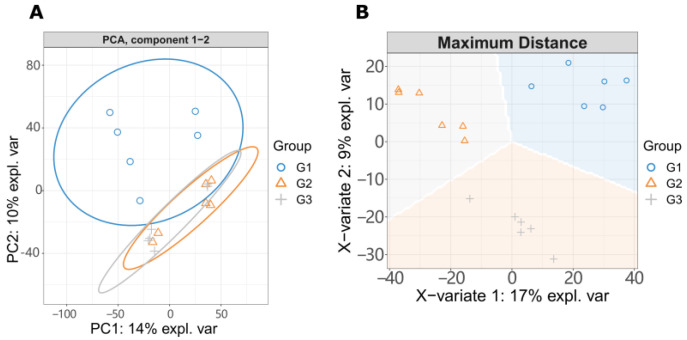
LC-MS/MS detected ions analysis. (**A**) PCA analysis. G1, tomato seeds soaking in water; G2, tomato seeds treated with 10 μg/L LGC; G3, tomato seeds treated with 100 μg/L LGC. (**B**) sPLS-DA analysis.

To observe the changes of metabolites with the increase of LGC concentration, we created three groups for metabolome investigation, including a blank (G1), low dose treated group (G2), and a high dose group (G3). All detected ions and identified metabolites (33 in total) were listed in the supplementary CSV files. We first analyzed the effect of LGC on the overall metabolome of tomato seedlings (*Solanum lycopersicum*, sly). As shown in Figure 3A, the PCA diagram indicated that LGC had a limited effect on the overall metabolome with a total difference of 24% in both vertical and horizontal directions. The sPLS-DA plot (Figure 3B) showed that the three groups of samples occupied relatively independent spaces, and the three groups of detected ions were distinguished. These differences of metabolome indicated can be explored further.

**Figure 4:**
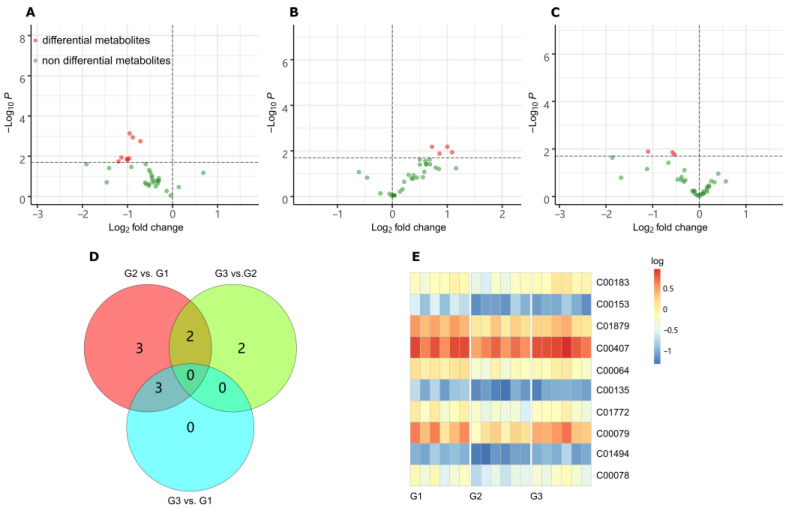
LC-MS/MS Metabolites analysis. (**A**) Volcano plot of differential metabolites (DMs) in the contrast of G2 vs. G1 (*p* < 0.02). (**B**) Volcano plot of DMs in the contrast of G3 vs. G2 (*p* < 0.02). (**C**) Volcano plot of DMs in the contrast of G3 vs. G1 (*p* < 0.02). (**D**) Venn plot of DM counts based on contrasts. (**E**) Heatmap of DMs variation. The vertical coordinate is the KEGG ID of DMs. Color represents the logarithm of the relative content.

The metabolites were identified based on full MS *m/z* and MS/MS spectra from the MSP database, whose entries were washed and limited to those KEGG compounds with important physiological roles (KEGG br08001, br08002, br08003, br08021). The mass accuracy was set as ±0.002 *m*/*z* for data collection and metabolite annotation. Based on this small database, 33 metabolites in total were identified, which were listed in the supplementary CSV file. The differential metabolites (DMs) were acquired by comparing G2 vs. G1, G3 vs. G2 and G3 vs. G1, respectively, as shown in the volcano Figure 4A–C. In total, 10 DMs were screened out with the thresholds of *p* < 0.02, as shown in the Venn plots (Figure 4D). Among them, two metabolites were associated with both comparisons of G2 vs. G1 and G3 vs. G2, which were l-phenylalanine (C00079) and l-tryptophan (C00078). Another three metabolites were related with both comparisons of G2 vs. G1 and G3 vs. G1, which were nicotinamide (C00153), l-pyroglutamic acid (C01879) and glutamine (C00064), respectively.

The changes of the 10 DMs in the three groups were summarized in the heatmap diagram in Figure 4E, seven of which are amino acids. When G2 was compared to G1, the most significant changes were phenylalanine (KEGG ID, C00079), nicotinamide (C00153), tryptophan (C00078), and trans-2-hydroxycinnamate (C01772). They have down-regulated more than 2.0-fold. While G3 was compared to G2, the expression levels of four amino acids (phenylalanine, valine, tryptophan, and isoleucine) increased significantly (more than 2.0-fold). The most increased amino acid was l-phenylalanine, which was also the most significantly decreased amino acid in the other contrast group G3 vs. G1.

### 2.3. Pathways Responding to LGC Regulation

**Figure 5:**
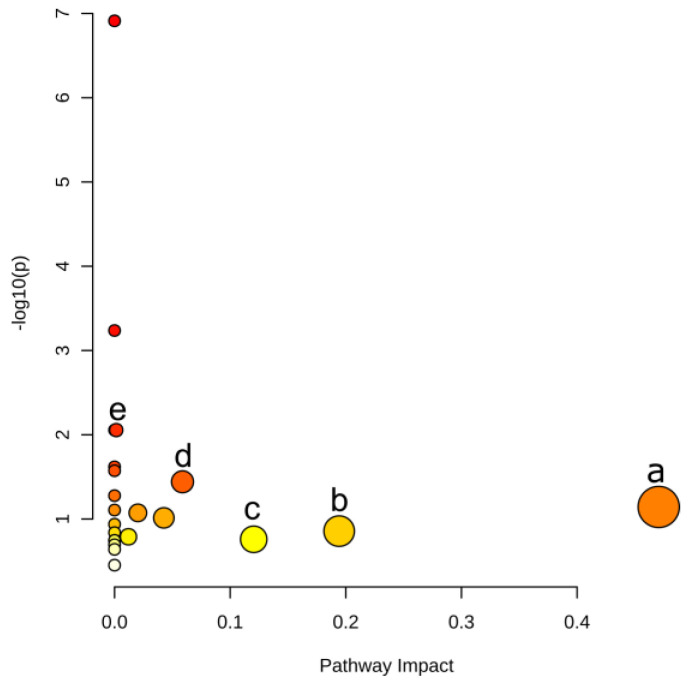
Pathway enrichment based on DMs. Points represent pathways, where size and color are correlated with the pathway impact. (**a**) Phenylalanine metabolism, (**b**) Alanine, aspartate and glutamate metabolism, (**c**) Tryptophan metabolism, (**d**) Phenylpropanoid biosynthesis, (**e**) Phenylalanine, tyrosine and tryptophan biosynthesis.

The DMs related pathways were enriched by MetaboAnalyst 5.0 associated with the KEGG database. There is no tomato (sly) option in MetaboAnalyst. We therefore used Arabidopsis data for pathway enrichment since both sly and Arabidopsis are Eudicots plants. As shown in Figure 5, significantly related metabolic pathways were gathered. Five pathways were prominent according to the pathway impact and t-test *p* values. These five pathways were phenylalanine metabolism, alanine, aspartate and glutamate metabolism, tryptophan metabolism, phenylpropanoid biosynthesis and phenylalanine, tyrosine and tryptophan biosynthesis, information for which are shown in Table 1.

**Figure 6:**
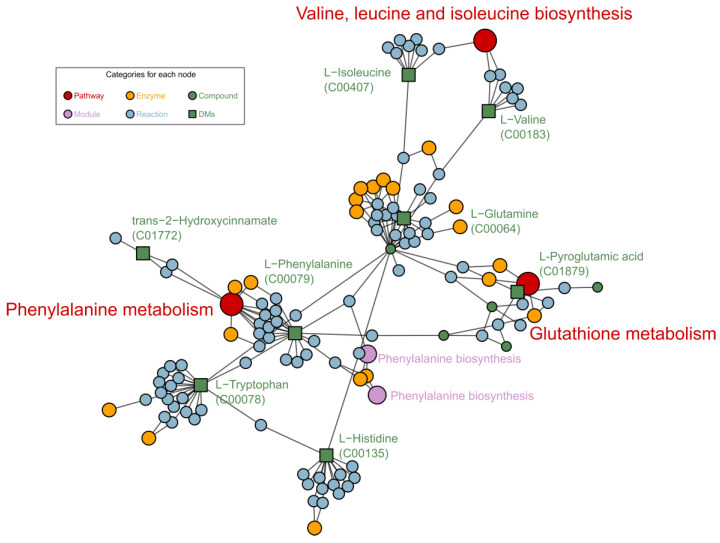
Metabolic pathways generated by FELLA, enriched based on KEGG sly data.

Enrichment analysis of FELLA (an R package) encompasses the closely associated pathways or metabolic modules to facilitate the observation of the overall metabolic response. We applied FELLA to enrich related reactions, enzymes, and pathways to the DMs. Related hit DMs changed significantly among the groups G1, G2 and G3 as shown in Supplementary Table S2. Phenylalanine metabolism, valine, leucine and isoleucine biosynthesis and glutathione metabolism pathways were enriched as shown in Figure 6. Phenylpropane metabolism is one of the most important plant secondary metabolic pathways, producing a variety of metabolites such as lignans. Valine, leucine and isoleucine biosynthesis responds differently when plants experience abiotic stresses during growth, and valine and leucine concentrations increase when plants are subjected to drought stress [16]. Glutathione metabolism is also essential for plant growth, especially in the regulation of ROS [17,18].

### 2.4. Phenotype Verification

**Figure 7:**
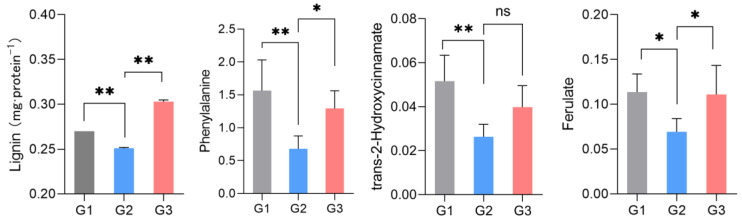
Lignin, phenylalanine, 2-hydroxycinnamate and ferulate expression regulated by LGC. * (*p* < 0.05), ** (*p* < 0.01), *n* = 6. ns = no significant difference.

Lignin is one of the signature metabolites of the above metabolic pathways and is easily measured. We therefore tested our conjecture by measuring the amount of lignin. As shown in Figure 7, the lignin expression decreased significantly in the G2 group but increased in the G3 group with increasing concentrations of LGC. This trend is consistent with the phenylalanine, ferulate and 2-hydroxycinnamate variations in metabolome analysis.

## 3. Discussion

### 3.1. LGC Regulation on Plant Growth

Bioactivity investigations showed that steroidal saponins have antimicrobial and cytotoxic activity [19]. Here, we focused on their effects on the growth and metabolism of tomato. The growth regulation effect on seeds is evaluated by germination percentage (GP), germination energy (GE) [20] and germination index (GI) [21]. GP responds to the viability of a population of seeds. Both GE and GI are indicators of seed vigor. GE is applied to evaluate the speed and uniformity of seedling emergence, while GI reflects GP and root length. The growth regulation effect on seedlings is evaluated by measurement of root length, plant length, fresh weight and dry weight. Both LGC and SA affected the seed germination and seedling growth of tomato. Compared to SA, LGC promotes better effects at a lower concentration (Figure 2) because LGC possesses more of the higher solubility glycosyl moiety that is more easily absorbed and transported into plants.

### 3.2. Metabolome Response to LGC

Since LGC showed better regulation effects on plant growth, it was selected to carry out a metabolism analysis to investigate the metabolic mechanism for regulation. The metabolome represents the whole metabolites variation within a biological system [22]. Compared to genomics and proteomics, metabolomics accurately and directly reflects the living organism’s response to external stimulations [23], and metabolites are usually used as effective marks to evaluate biological phenotypes and states.

Metabolomes are generally measured by NMR or LC-MS/MS. The NMR methodology provides a simple procedure for sample preparation and more characteristic information related to chemical structures. In contrast, LC-MS/MS affords higher sensitivity and accuracy for quantification. The tandem mass spectrometry (MS/MS or MS^n^) can afford much information on thousands of chemicals from one measurement [24,25]. We therefore preferred to apply an LC-MS/MS method to monitor the changes of metabolites due to the high sensitivity. To increase the accuracy of chemical structures annotation, we joined the public databases of MS/MS reference database from MS-Dial [26] and removed unrelated records such as synthetic compounds. This ‘data-washing’ can enhance the reliability of chemical structure annotation. 

Currently, metabolite identification is classified into four levels, namely identified compounds, putatively annotated compounds, putatively characterized compound classes, and unknown compounds. We annotated metabolites at level 2 according to the requirements of the article [27].

We chose metabolomes to explore the response to LGC within the plant. To identify metabolites directly related to LGC as much as possible, we set up three groups of experiments with blank, low dose of LGC and high dose of LGC. Metabolites that varied in pairwise contrasts (G2 vs. G1, G3 vs. G2 and G3 vs. G1) were filtered out as DMs. A total of 10 DMs were selected for further pathway enrichment and metabolomic investigations. The DMs contained seven amino acids, two phenylpropanoids and nicotinamide. The metabolome showed different response trends to low and high dose treatment of LGC. The expression level of four metabolites decreased in the G2 group (low dose) and then increased in the G3 group (high dose). They were phenylalanine (Phe), nicotinamide (VB3), tryptophan (Trp), and trans-2-hydroxycinnamate.

### 3.3. Metabolic Mechanisms of LGC Regulation on Plant Growth

Among the pathways illustrated in Figure 5 and Figure 6, the phenylalanine metabolism pathway was the most affected pathway, based on path impact and statistical *p* values. The phenylalanine pathway connects four other metabolic pathways as listed in Table 1. Among these pathways, the phenylpropanoid metabolism lies at the boundary between primary and secondary metabolism [28] and allows the transfer of carbon sources from phenylalanine to the three monolignols as well as other phenolic compounds [29]. In our current investigation, l-phenylalanine and ferulate changed significantly with increasing LGC concentrations. When LGC acted as a growth promoter in the G2 group, l-phenylalanine and ferulate decreased 1.64~2.30-fold, whereas, in the G3 group, LGC inhibited seedling growth, and the concentrations of the metabolites noted increased 1.65~2.13-fold. Lignin is generated from l-phenylalanine via phenylpropanoid biosynthesis. The increased level of l-phenylalanine and phenylpropanoid ferulate in G3 invoked the synthesis of lignin, which inhibited root elongation and the seedling growth of tomato. Thus, it was necessary to verify whether LGC affects lignin expression in tomato seedlings.

### 3.4. LGC Regulation on Lignin

Lignin is the final product of phenylpropanoid metabolism in plants, and it is essential for plant growth. The metabolomic analysis indicated that phenylalanine and ferulate were related to the phenylpropanoid metabolism, which was affected by LGC regulation. In the current study, we carried out verification to confirm that LGC can cause changes in lignin. Lignin levels in seedlings can be quantified by A_280_ in UV spectrophotometry [30]. These results indicated that high dose LGC up-regulated phenylalanine metabolism and the phenylpropanoid pathway and increased lignin expression levels, which would solidify the cell wall and inhibit root growth [31]. On the contrary, a low concentration of LGC down-regulated the metabolism of phenylalanine and the synthesis pathway of precursors of lignans, phenylpropanoid, which would inhibit the expression level of lignans and promote plant growth. Elsewhere it has been noted that overexpressed phenylalanine can affect the lignin content of plants and contribute prominently to plant growth [28]. Thus, our result suggested that LGC controlled lignin synthesis by regulating phenylalanine.

## 4. Materials and Methods

### 4.1. General

NMR spectra were recorded on an AVANCE III spectrometer (400 MHz). The solvent residue was referred to by the chemical shifts (in ppm). Coupling constants (*J*) were recorded in Hertz. LC-MS/MS data were read on an AB Sciex Triple TOF 5600+ instrument (AB Sciex Pte. Ltd., Framingham, MA, USA). Tomatoes were grown in an artificial climate chamber. Plant photos were scanned by an Epson Perfection V370 Photo. Chemicals were bought from Energy Chemical Co. Ltd. (Shanghai, China) without further purification before use.

### 4.2. Preparation and Identification of SA and LGC

SA and LGC were isolated and purified following a reported method [15]. In brief, dried powder (4.8 kg) of *Smilax scobinicaulis* rhizome was extracted with 75% ethanol at reflux for 8 h. The extract was concentrated under reduced pressure to yield a crude residue which was extracted with EtOAc and n-BuOH, respectively. The n-butanol extracted fraction was separated on macroporous adsorbent resin D101, silica gel column and purified on RP-18 column chromatography to afford SA (26 mg) and LGC (100 mg). Their chemical structures were identified by comparing NMR and high-resolution (HR) MS data with reported ones.

Smilaxin A (SA). ^1^H NMR (400 MHz, DMSO-*d*_6_) *δ* 5.00–4.91 (m, 2H), 4.52 (dd, *J* = 12.3, 4.6 Hz, 1H), 4.21 (dd, *J* = 11.1, 6.7 Hz, 2H), 3.70–2.98 (m, 9H), 2.15–2.03 (m, 1H), 1.85 (td, *J* = 13.9, 12.7, 5.8 Hz, 3H), 1.79–1.63 (m, 3H), 1.63–1.52 (m, 2H), 1.52–1.44 (m, 1H), 1.41–1.11 (m, 6H), 0.91 (d, *J* = 6.9 Hz, 2H), 0.77–0.70 (m, 4H), 0.66 (s, 2H); ^13^C NMR (100 MHz, DMSO-*d*_6_) *δ* 210.43, 108.89, 103.97, 100.88, 80.47, 77.08, 76.04, 75.92, 73.83, 73.02, 70.95, 70.6, 68.64, 67.72, 66.39, 65.25, 62.21, 55.99, 55.65, 52.93, 46.39, 41.54, 40.9, 40.81, 37.16, 36.44, 31.63, 31.35, 30.26, 29.03, 28.92, 26.56, 21.3, 17.55, 16.56, 15.08, 13.28. HR ESI-MS *m/z* Full MS [M + CH_3_COO]^−^ calcd for C_40_H_63_O_15_^−^ 783.41724, found 783.4166 (error 0.82 ppm).

Laxogenin C (LGC). ^1^H NMR (400 MHz, MeOD) *δ* 4.56 (d, *J* = 7.9 Hz, 1H), 4.48–4.38 (m, 2H), 4.35 (d, *J* = 7.1 Hz, 1H), 4.15 (dd, *J* = 11.1, 1.8 Hz, 1H), 3.96–3.78 (m, 4H), 3.77–3.34 (m, 12H), 3.34–3.17 (m, 8H), 2.38 (dd, *J* = 13.1, 3.0 Hz, 1H), 2.26 (dd, *J* = 13.0, 4.7 Hz, 1H), 2.15 (t, *J* = 12.5 Hz, 1H), 2.08–1.88 (m, 5H), 1.82 (d, *J* = 6.5 Hz, 1H), 1.81–1.67 (m, 4H), 1.67–1.47 (m, 5H), 1.45 (t, *J* = 1.9 Hz, 1H), 1.43 (t, *J* = 5.0 Hz, 2H), 1.41–1.23 (m, 5H), 0.99 (d, *J* = 6.9 Hz, 3H), 0.85–0.77 (m, 8H); ^13^C NMR (100 MHz, MeOD) *δ* 211.98, 109.15, 103.90, 103.04, 100.82, 80.53, 79.06, 77.11, 76.46, 74.93, 73.74, 73.64, 73.42, 73.03, 70.98, 70.07, 68.42, 66.94, 66.46, 65.73, 62.30, 61.00, 56.08, 56.00, 53.38, 48.24, 46.08, 41.49, 40.75, 40.71, 39.22, 37.44, 36.28, 31.06, 31.00, 30.03, 28.56, 28.47, 25.91, 21.04, 16.10, 15.44, 13.45, 12.04. HR ESI-MS *m/z* Full MS [M + CH_3_COO]^−^, C_46_H_73_O_18_^−^, calcd. 945.47007, found 945.4689 (error 1.24 ppm).

### 4.3. Plant Culture and Growth Evaluation

Following a reported protocol with slight modification [32], tomato (*Lycopersicon esculentum* Mill.) seeds were put into 10% H_2_O_2_ for 5 min, followed by washing in sterilized distilled water six times. The seeds were then put into clear water, SA (1, 5, 10, 50, 100 μg/L) and LGC (1, 5, 10, 50, 100 μg/L) respectively and soaked 24 h without light. After washing in sterilized distilled water, 20 seeds of each biological treatment were sown in a petri dish covered with two filter papers, and transferred in a constant temperature incubator (for each biological treatment, seeds were sown in petri dishes and transferred at a constant temperature incubator, with each dish containing 20 seeds and covered with two filter papers) (25 °C, light/dark = 12/12, light intensity 600 lx, humidity 80%) for 5 d. The above experiments were repeated three times.

The number of germinated seeds was counted daily. Seeds with at least 2.0 mm of radicle were considered to be germinated. Germination percentage, germination energy and germination index were calculated according to the following formula:

(1) $$\mathrm{Germination}\;\mathrm{percentage}\;\left(\%\right)=\left[\frac{\left(\mathrm{number}\;\mathrm{of}\;\mathrm{seeds}\;\mathrm{germinated}\;\mathrm{on}\;\mathrm{the}\;5\mathrm{th}\;\mathrm{day}\right)}{\left(\mathrm{total}\;\mathrm{number}\;\mathrm{of}\;\mathrm{seeds}\;\mathrm{tested}\right)}\right]\times 100\%\;$$

(2) $$\mathrm{Germination}\;\mathrm{energy}\;\left(\%\right)=\left[\frac{\left(\mathrm{number}\;\mathrm{of}\;\mathrm{germinated}\;\mathrm{seeds}\;\mathrm{on}\;\mathrm{the}\;\sec\mathrm{ond}\;\mathrm{day}\right)}{\left(\mathrm{total}\;\mathrm{number}\;\mathrm{of}\;\mathrm{seeds}\right)}\right]\times 100\%$$

(3) $$\mathrm{Germination}\;\mathrm{index}\;=\left[\frac{\left(\mathrm{seed}\;\mathrm{germination}\;\times \;\mathrm{root}\;\mathrm{length}\;\mathrm{of}\;\mathrm{the}\;\mathrm{treatment}\right)}{\left(\mathrm{seed}\;\mathrm{germination}\;\times \;\mathrm{root}\;\mathrm{length}\;\mathrm{of}\;\mathrm{the}\;\mathrm{control}\right)}\right]\times 100\%$$

Root length, plant length, fresh weight and dry weight of tomato seedlings were measured and recorded after 5 d culture. The seedlings were scanned on an Epson scanner and the root and seedling length were quantified using ImageJ software. The averages and deviations of germination energy, germination index and plant weight were counted on seedlings in each culture dish.

### 4.4. LC-MS/MS Profiling of Plant Metabolome

We set a blank (G1) and two treated groups containing 5μg/L (G2) and 100μg/L (G3) of LGC, respectively, to investigate the variation of tomato metabolites. Each group had six biological replicates, which were collected with 1.0 g fresh seedlings (including root and stem) after being cultured for five days. The collections were then immediately frozen with liquid N_2_ and ground into powder. Samples were extracted with 5 mL MeCN and sonicated for 30 min. The mixture was centrifuged at 12,000× *g* rpm at 25 °C for 10 min. The supernatant was filtered through a 0.22 µm filter and stored at −80 °C before LC-MS analysis.

5 µL of each sample was injected for analysis on a UPLC-qTOF-ESIMS system. The metabolites were separated on a C_18_ column (Shim-pack XR-ODS, 2.0 mm × 100 mm) and eluted with a gradient MeCN (0%~100%, with 0.1% AcOH, 0.3 mL/min) for 18 min. The full MS was scanned from 50 *m*/*z* to 1250 *m*/*z* in positive mode. The MS/MS data were collected using the data-independent acquisition (SWATH) method. The collision energy was set at 35 V with a spread of 15 V.

### 4.5. Data Analysis and Visualization

Data procedure. The LC-MS WIFF raw data files were converted to ABF format in the Free software Reifycs ABF converter. The ABF files were then imported into MS-Dial (version 4.7.0) to explore the MS data. Each duplicate piece of data was aligned and normalized according to the TIC and the internal standard (6-bromo-4-hydroxycoumarin). According to the reported procedure, the mass accuracy of MS1 and MS2 in data collection was set as 2 mDa and 5 mDa [33,34]. 

Putative annotation. We used the MS-DIAL metabolomics MSP spectral kit (all public MS/MS spectra VS15) for metabolite annotation. However, the MS-Dial MSP database contains too many unrelated compounds, such as synthetic compounds and plasma components. We cleaned MSP data to exclude compounds not in the KEGG database using a python script. This procedure can avoid the apparent wrong annotation of synthetic products. The annotation procedure was based on average *m/z* (2 mDa tolerance) and MS/MS spectrum (5 mDa tolerance). All the detailed thresholds and settings used in MS-Dial for the data procedure and annotation were listed in Supplementary Table S1.

Visualization. The LC-MS data analysis and visualization were performed according to our previously reported method [35]. The alignment data (including MS features, metabolite annotations, and normalized peak areas) was then exported as a CSV file for further visualization. The PCA and sPLS-DA were analyzed and visualized using the R package mixOmics. Statistical *p*-values were calculated using the one way test function in R. The R package ggplot2 was used to draw the volcano plots, and the R package ggvenn was used to create the Venn plots. The DMs were obtained according to the contrast of G1 vs. G2 and G2 vs. G3 with the threshold *p*-value < 0.02. KEGG enrichment and pathway were carried out by the online tool MetaboAnalyst 5.0 (https://www.metaboanalyst.ca, accessed on 25 August 2021) associated with the KEGG metabolic pathway database of Arabidopsis thaliana. The pathway relationship was mapped using an R package FELLA (version 1.14.0) [36,37] associated with the KEGG data of *Solanum lycopersicum* (accessed on 12 January 2022).

### 4.6. Lignin Quantification 

To further verify the effect of LGC on tomato lignin, we cultured tomatoes in the same way and determined the lignin content by UV spectrophotometry. The method of lignin extraction from five-day-old seedlings referred to the method [30] with slight modifications. A total of 0.5 g fresh sample was ground into a homogenate with 95% ethanol, centrifuged at 4500× *g* rpm for 10 min, and the precipitate was rinsed three times with 95% EtOH, then three times with 1:1 (*v/v*) of EtOH and n-hexane. The dried plant material was extracted with 0.5 mL of 25% bromoacetyl in AcOH under 70 °C for 30 min. The reaction was terminated by adding 0.9 mL NaOH (2 mol/L). Then 5 mL AcOH and 0.1 mL hydroxylamine (7.5 mol/L) was added to the mixture. After centrifuging at 4500× *g* rpm for 5 min, a further 3.0 mL AcOH was added to the mixture. The lignin concentration was quantified by the absorption at 280 nm.

## 5. Conclusions

LGC is a natural derivate of plant endogenous hormones and is widely used as an eco-friendly regulator for plant growth in agriculture. The variation of plant components is highly related to the quality and nutrition of crops or the drug efficacy of traditional herbs. We herein applied LC-MS/MS to investigate the metabolome response to the regulator and revealed 10 DMs highly related to LGC regulation. The most significant changes were l-phenylalanine and a phenylpropanoid ferulate, precursors for lignin biosynthesis. Pathway enrichment analysis showed the effects that LGC had on phenylalanine and phenylpropanoid biosynthesis pathways related to lignin accumulation. These findings provided a metabolomic aspect on the plant hormone derivates and revealed the affected metabolites. It is anticipated that further studies on agrichemical development will provide eco-friendly and efficient regulators for plant growth control and quality improvement.

## Figures and Tables

**Table 1:** Matched features in KEGG pathways analysis.

Pathway	–logP	Impact	Matched Features
Phenylalanine metabolism	1.14	0.470	l-phenylalanine (C00079)
Alanine, aspartate and glutamate metabolism	0.86	0.194	l-glutamine (C00064)
Tryptophan metabolism	0.76	0.120	l-tryptophan (C00078)
Phenylpropanoid biosynthesis	1.44	0.059	l-phenylalanine (C00079); ferulate (C01494)
Phenylalanine, tyrosine and tryptophan biosynthesis	2.05	0.002	l-phenylalanine (C00079); l-tryptophan (C00078)

## Data Availability

Data is contained within the article and supplementary material.

## Supplementary Materials

The following supporting information can be downloaded at: https://www.mdpi.com/article/10.3390/ijms23062990/s1.
