# Supplementary material for: Metabolomics Mechanism and Lignin Response to Laxogenin C, a Natural Regulator of Plants Growth
Source: Int J Mol Sci. 2022 Mar 10;23(6):2990. doi: 10.3390/ijms23062990 (PMC8951225; doi:10.3390/ijms23062990)
Supplement: Supplementary file 1 [file ijms-23-02990-s001.zip › Supplementary data_v7.pdf]

Metabolomics Mechanism and Lignin Response to Laxogenin C, a Natural Regulator of Plants Growth

Yu-Chan Deng, Jia-Qi Wang, Zhao-Ju Zhu, Si-Pei Ren, Cun-Li Zhang,* Qiang Zhang*

Shaanxi Key Laboratory of Natural Products & Chemical Biology, College of Chemistry & Pharmacy, Northwest A&F University, Yangling 712100, China

* Corresponding author:

Qiang Zhang, E-mail: zhangq@nwsuaf.edu.cn

Cun-Li Zhang, E-mail: cunli_zhang@nwsuaf.edu.cn

Contents

Figure S1. Daily germination of seeds treated with LGC and SA 2

Figure S2. Tomato seedlings treated with different solutions. 3

Figure S3. ^{1}H NMR spectra of LGC in MeOD 4

Figure S4. ^{13}C NMR spectra of LGC in MeOD 5

Figure S5. HR ESI MS of LGC 6

Figure S6. ^{1}H NMR spectra of SA in DMSO-d_6 7

Figure S7. ^{13}C NMR spectra of SA in DMSO-d_6 8

Figure S8. HR ESI MS of SA 9

Figure S9. Measurement and Reference MS/MS Spectra Comparison 10

Table S1. Thresholds and Settings in MS-Dial 12

Table S2. Hit DMs related to plant growth regulation 13

Figure S1. Daily germination of seeds treated with LGC and SA

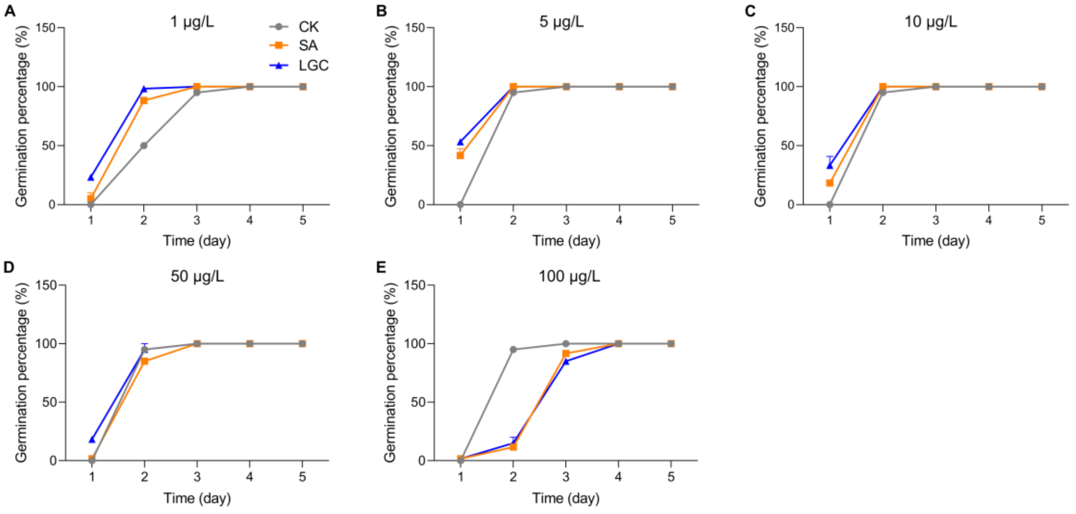

Figure S2. Tomato seedlings treated with different solutions.

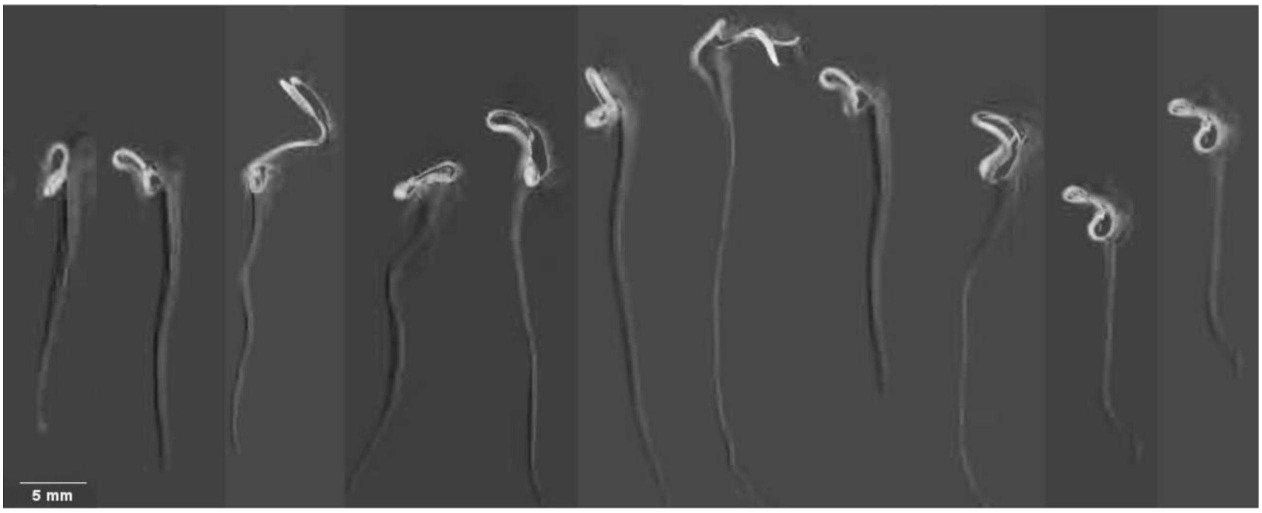

CK	1 μg/L SA	1 μg/L LGC	5 μg/L SA	5 μg/L LGC	10 μg/L SA	10 μg/L LGC	50 μg/L SA	50 μg/L LGC	100 μg/L SA	100 μg/L LGC

Figure S3. ^{1}H NMR spectra of LGC in MeOD

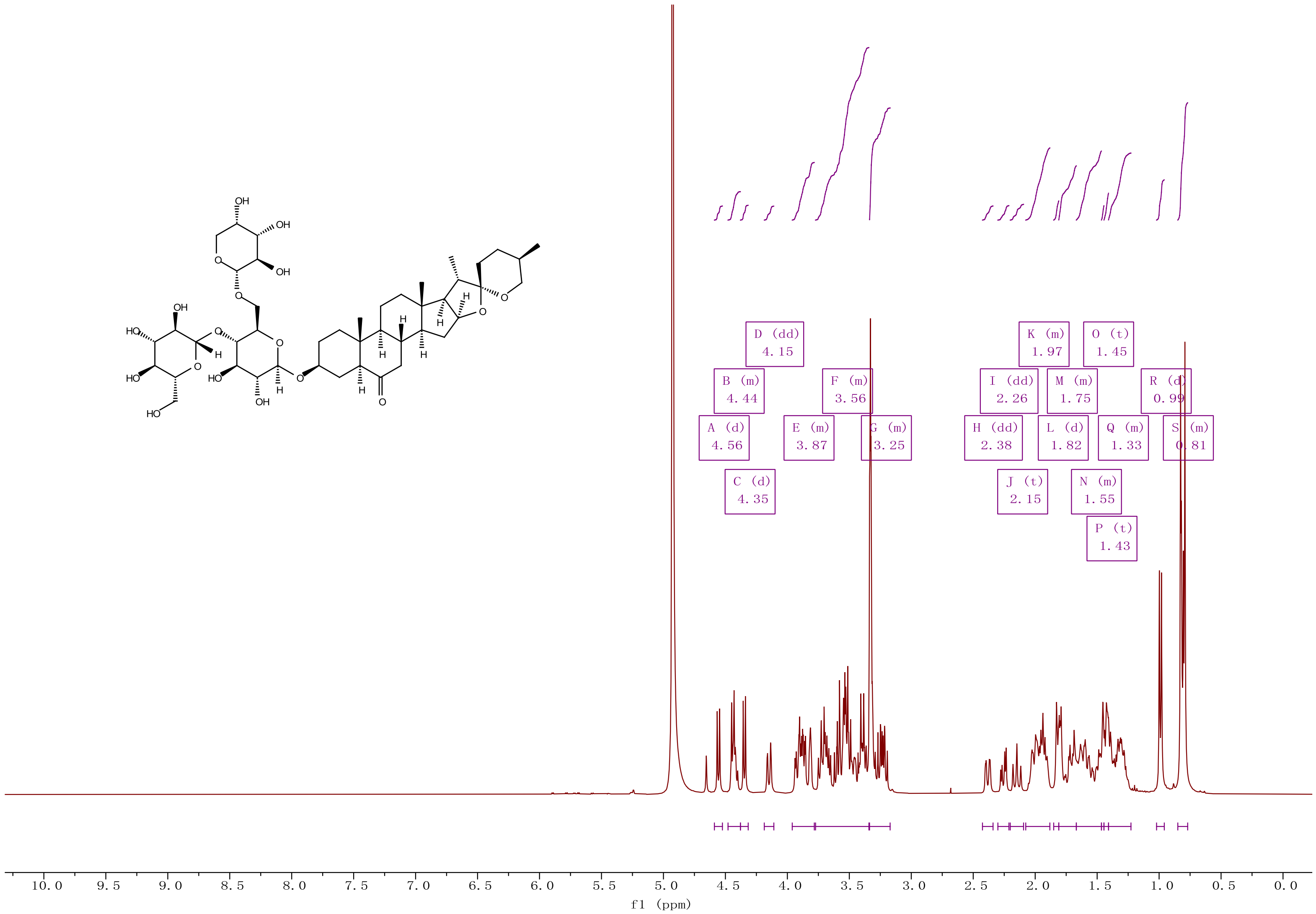

Figure S4. ^{13}C NMR spectra of LGC in MeOD

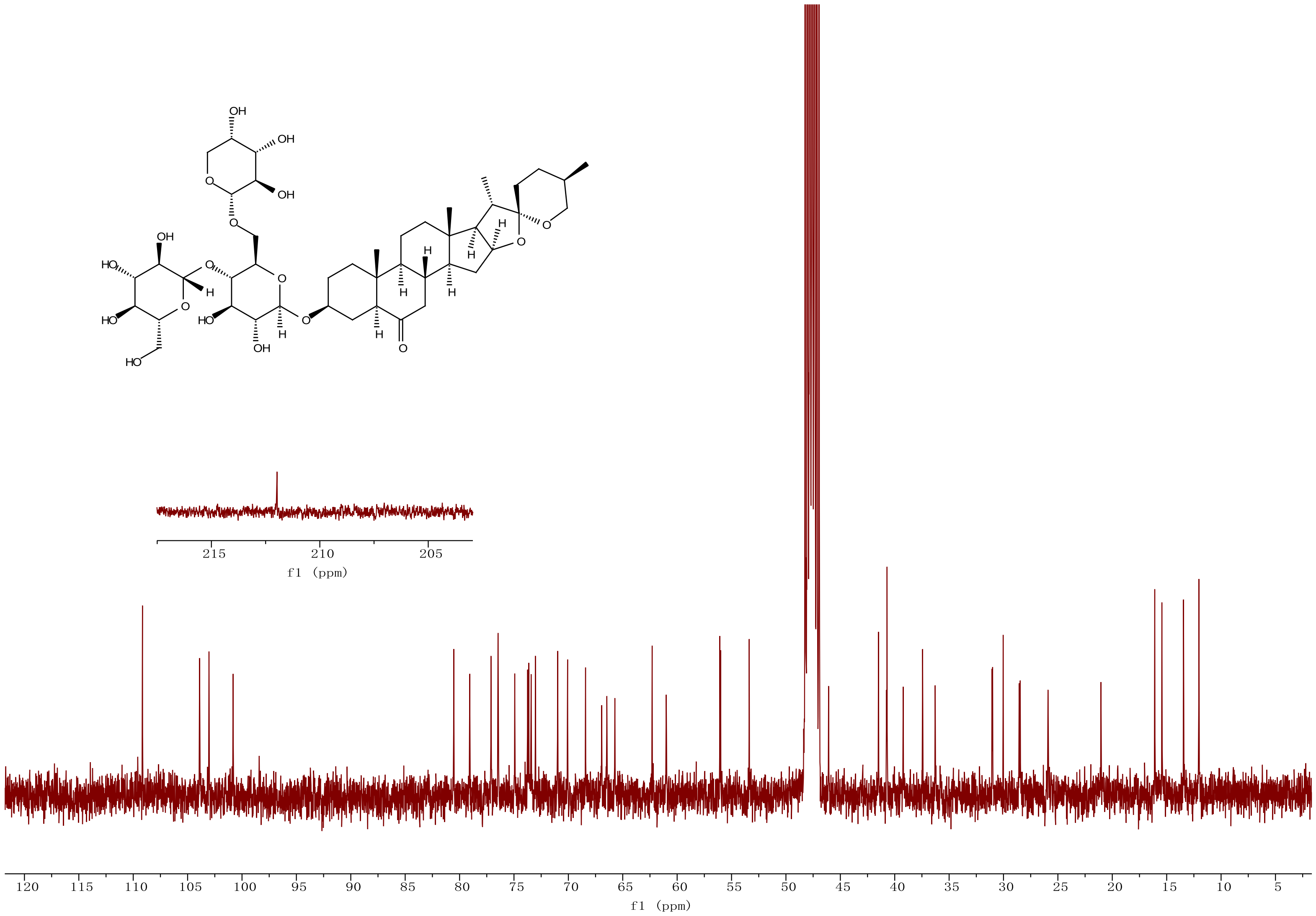

Figure S5. HR ESI MS of LGC

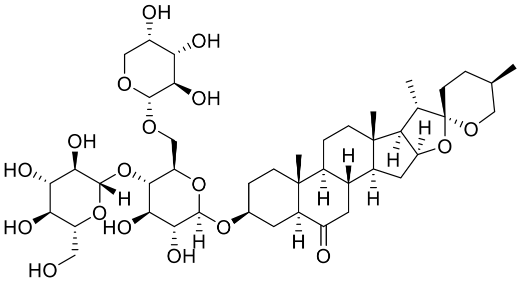

LGC Chemical Formula: $C_{44}H_{70}O_{18}$

$[M+CH_3COO]^-$, $C_{46}H_{73}O_{18}^-$, calcd. 945.47007, found 945.4689 (1.24 ppm)

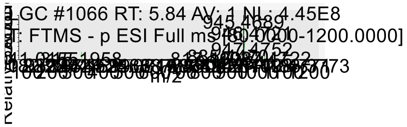

Figure S6. ^{1}H NMR spectra of SA in DMSO-d_6

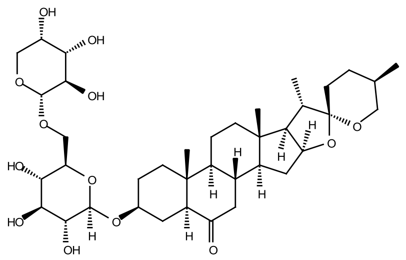

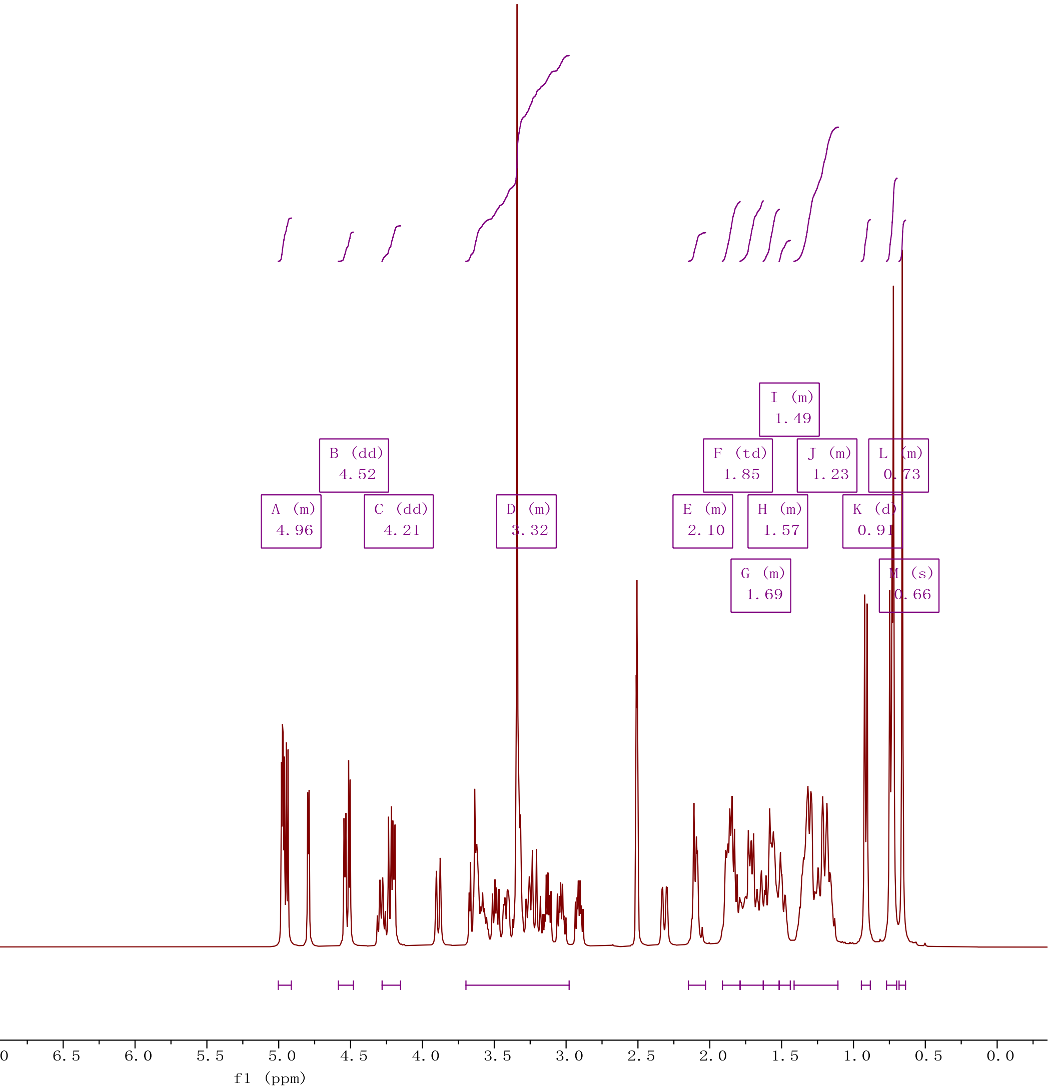

Figure S7. ^{13}**C NMR spectra of SA in DMSO-*d*$_6$**

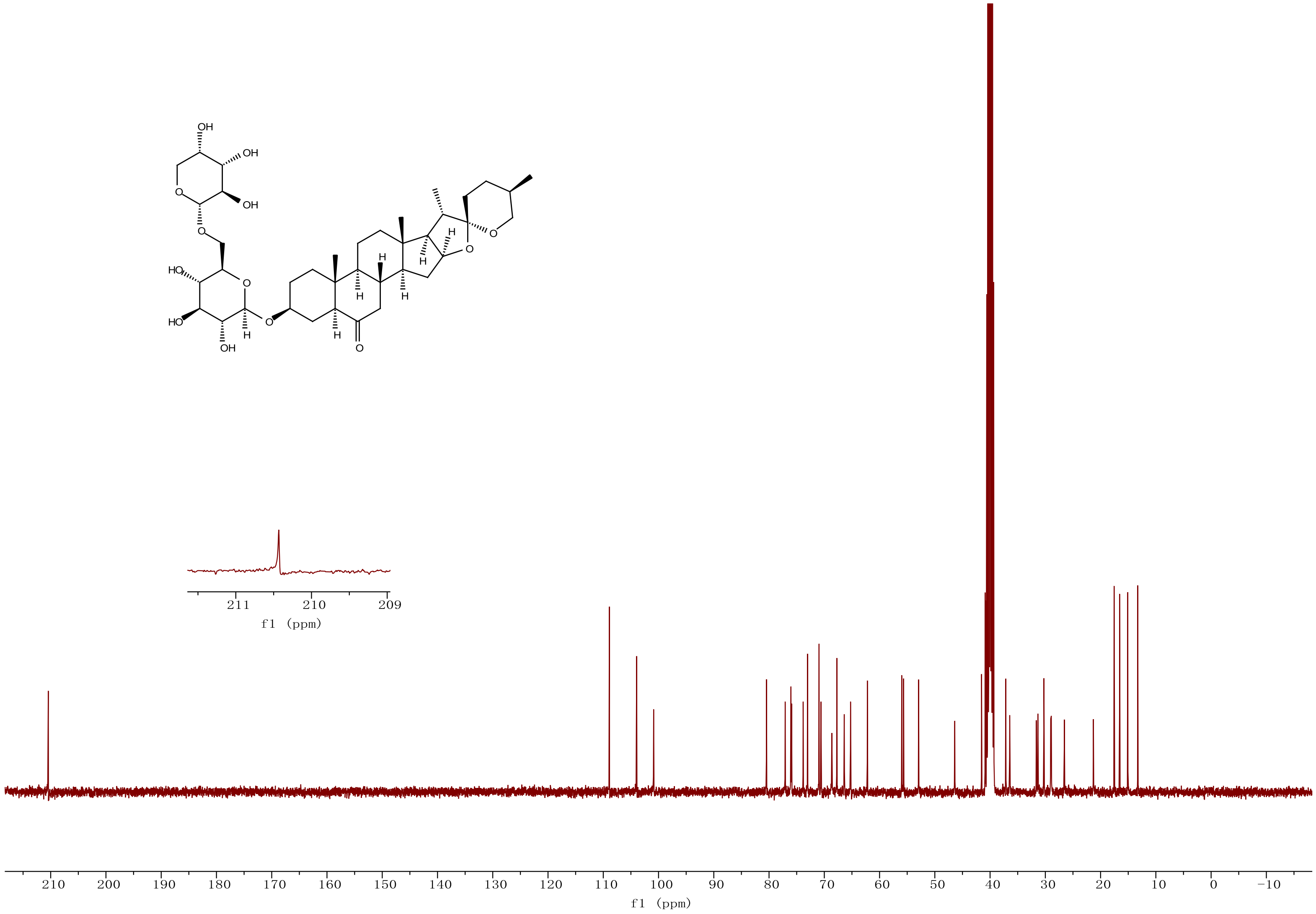

Figure S8. HR ESI MS of SA

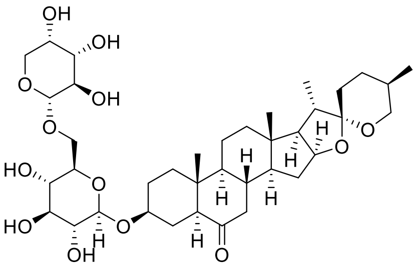

SA Chemical Formula: $C_{38}H_{60}O_{13}$

$[M+CH_3COO]^-$, $C_{40}H_{63}O_{15}^-$, calcd. 783.41724, found 783.4166 (0.82 ppm)

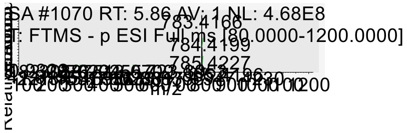

Figure S9. Measurement and Reference MS/MS Spectra Comparison

Alignment ID 181 2-Hydroxycinnamate

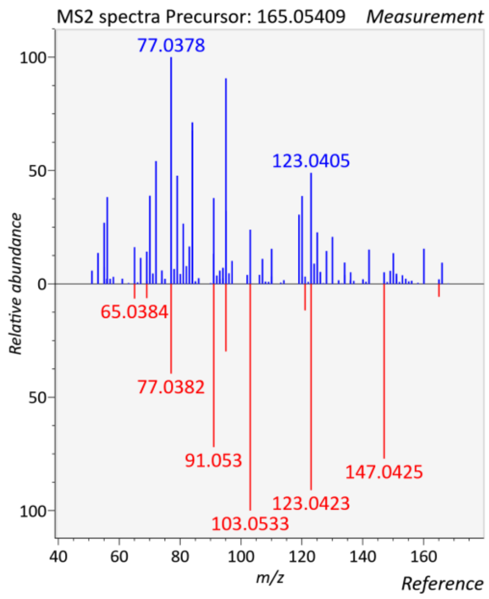

Alignment ID 184,

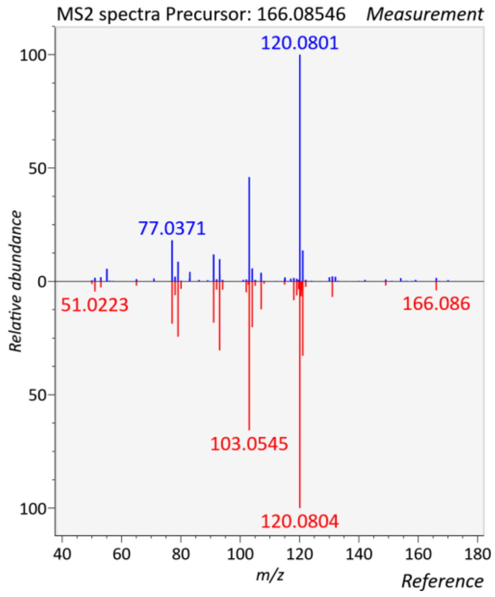

Alignment 220, Ferulate

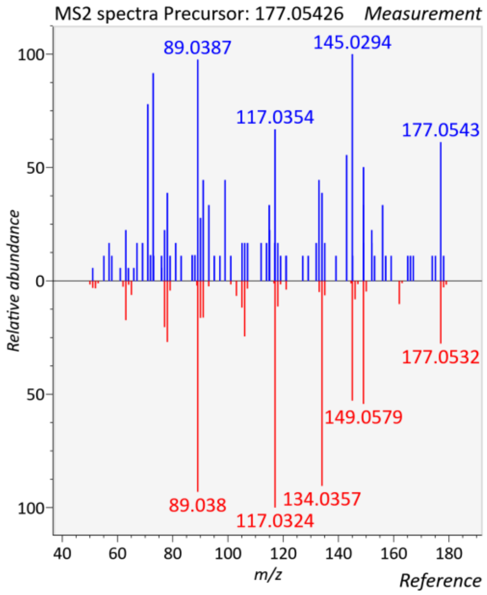

Table S1. Thresholds and Settings in MS-Dial

MS-DIAL ver. 4.80

#Centroid parameters
MS1 tolerance 0.002
MS2 tolerance 0.005

#Isotope recognition
Maximum charged number 2

#Peak detection parameters
Smoothing method LinearWeightedMovingAverage
Smoothing level 3
Minimum peak width 5
Minimum peak height 500

#Peak spotting parameters
Mass slice width 0.05
Exclusion mass list (mass & tolerance)

#Deconvolution parameters
Sigma window value 0.5
MS2Dec amplitude cut off 0
Exclude after precursor True
Keep isotope until 5
Keep original precursor isotopes False

#MSP file and MS/MS identification setting
Accurate mass tolerance (MS1) 0.002
Accurate mass tolerance (MS2) 0.005
Identification score cut off 60

#Adduct ion setting
[M+H]+
[M+Na]+
[2M+H]+
[2M+Na]+

#Alignment parameters setting
Retention time tolerance 0.1
MS1 tolerance 0.01
Retention time factor 0.5
MS1 factor 0.5

Table S2. Hit DMs related to plant growth regulation

Alignment ID	KEGG ID	Metabolite name	Formula	Rt(min)	Average M/Z	log2 (G2/G1)	p (G2, G1)	log2 (G3/G2)	P (G3, G2)	log2 (G3/G1)	P (G3, G1)
38	C00183	L-Valine	$C_5H_{11}NO_2$	1.024	118.08684	/	/	1.00	0.07	/	
49	C00153	Niacinamide	$C_6H_6N_2O$	1.024	123.05451	-1.13	0.012	/		-1.10	0.013
63	C01879	L-Pyroglutamic acid	$C_5H_7NO_3$	1.024	130.04842	-0.88	0.001	/		-0.53	0.017
68	C00407	L-Isoleucine	$C_6H_{13}NO_2$	1.081	132.10136	/	/	0.72	0.07	/	
110	C00064	L-Glutamine	$C_5H_{10}N_2O_3$	1.024	147.07642	-0.95	0.001	/		-0.58	0.014
152	C00135	L-Histidine	$C_6H_9N_3O_2$	1.024	156.07664	-0.96	0.013	/		/	
181	C01772	trans-2-Hydroxycinnamate	$C_9H_8O_3$	1.081	165.0546	-1.00	0.015	/		/	
184	C00079	L-Phenylalanine	$C_9H_{11}NO_2$	1.195	166.08531	-1.20	0.018	1.09	0.12	/	
220	C01494	trans-Ferulic acid	$C_{10}H_{10}O_4$	3.127	177.05426	-0.71	0.002	/		/	
289	C00078	Tryptophan	$C_{11}H_{12}N_2O_2$	1.195	205.09776	-1.01	0.014	0.86	0.13	/	

Note: /, no significant variation.
